# Administration of follitropin alfa and lutropin alfa combined in a single injection: a feasibility assessment

**DOI:** 10.1186/1477-7827-7-48

**Published:** 2009-05-18

**Authors:** Rita Agostinetto

**Affiliations:** 1 Merck Serono S.p.A., Via Luigi Einaudi 11, 00012 Guidonia Montecelio, Rome, Italy

## Abstract

**Background:**

Gonadotrophins are routinely administered in assisted reproductive technology (ART) treatment protocols. Recombinant human follicle-stimulating hormone (r-hFSH; follitropin alfa) and recombinant human luteinizing hormone (r-hLH; lutropin alfa) can be administered individually or in a fixed combination. The ability to vary the FSH to LH dose ratio in a single injection without compromising the bioactivity of either gonadotrophin or generating losses of active principle is important for physicians and patients alike.

**Methods:**

This study investigated whether follitropin alfa (GONAL-f (R)), as lyophilized powder for reconstitution or solution from the GONAL-f (R) (filled-by-mass [FbM]) Prefilled Pen, could be used to reconstitute Pergoveris TM (follitropin alfa/lutropin alfa 150 IU/75 IU) lyophilized powder. In Ratio Groups 1 and 2, the r-hFSH:r-hLH ratio was 3:1; in Ratio Groups 3 and 4, the ratios of r-hFSH:r-hLH were 5:1 and 8:1, respectively. The protein content and bioactivity of each mixed solution were evaluated. The r-hFSH and r-hLH content was determined using reverse-phase high performance liquid chromatography. The biological activity of r-hFSH and r-hLH was assessed using the Steelman-Pohley and Van Hell in vivo bioassays in rats, respectively.

**Results:**

Follitropin alfa/lutropin alfa 150 IU/75 IU lyophilized powder could be successfully mixed with follitropin alfa 75 IU FbM solution that was either reconstituted from lyophilized powder or injected directly from the prefilled pen to create solutions with ratios of r-hFSH and r-hLH from 3:1 to 8:1. The measured content of r-hFSH and r-hLH corresponded favourably with the target protein content in Ratio Groups 1–4. The in vivo target and measured bioactivity of r-hFSH and r-hLH were also closely matched in all Ratio Groups.

**Conclusion:**

Follitropin alfa lyophilized powder or solution can be accurately mixed with follitropin alfa/lutropin alfa 150 IU/75 IU lyophilized powder to enable physicians to administer ratios of r-hFSH and r-hLH in the IU range from 3:1 to 8:1. Mixing of recombinant formulations offers flexibility for patients receiving follitropin alfa and lutropin alfa for ART protocols in clinical practice.

## Background

Gonadotrophins are routinely administered in many assisted reproductive technology (ART) treatment protocols. Follicle-stimulating hormone (FSH) induces the recruitment and development of ovarian follicles and luteinizing hormone (LH) increases oestradiol secretion by the follicles and is responsible for final oocyte maturation and subsequent ovulation [1]. Recombinant human FSH (r-hFSH; follitropin alfa) and LH (r-hLH; lutropin alfa) can be administered independently or in combination, in accordance with individual patient needs.

Various injection devices have been developed to simplify administration of gonadotrophins and allow patients to self-administer treatment [2]. Follitropin alfa (filled-by-mass [FbM]) is provided in ready-to-use, prefilled pens that are designed specifically for self-administration by patients [2,3]. The GONAL-f^® ^(FbM) Prefilled Pen is available in three multi-dose formulations (300, 450 and 900 IU) and does not require reconstitution or cartridge assembly into the injection device, thereby reducing the number of steps before injection and helping to ensure an accurate and correct dose [2,3]. Follitropin alfa (FbM) is also available in single (75 IU) and multidose (450 and 1050 IU) vials of lyophilized powder for reconstitution. The variety of commercially available preparations accommodates individualized dosing for diverse protocols for ovulation induction or controlled ovarian stimulation.

r-hFSH and r-hLH have been recently combined in a single product (Pergoveris™ [follitropin alfa/lutropin alfa 150 IU/75 IU]), thereby allowing administration of both gonadotrophins in a convenient single injection [4]. Follitropin alfa/lutropin alfa 150 IU/75 IU is indicated for the stimulation of follicular development in women who are infertile because of severe endogenous FSH and LH deficiencies [4].

An increasing number of physicians are requesting information on the feasibility of mixing the currently available recombinant gonadotrophin products. Therefore, we evaluated the protein content and *in vivo *bioactivity of follitropin alfa and lutropin alfa in rats after mixing follitropin alfa/lutropin alfa 150 IU/75 IU lyophilized powder with follitropin alfa as 75 IU FbM lyophilized powder or solution from the GONAL-f^® ^(FbM) Prefilled Pen.

## Methods

### Materials

Commercially-available formulations of recombinant gonadotrophins were tested: follitropin alfa/lutropin alfa 150 IU/75 IU [Pergoveris™] lyophilized powder; follitropin alfa (GONAL-f^®^) as 75 IU FbM lyophilized powder (in 3 mL glass vials) supplied by Laboratoires Serono S.A. (Aubonne, Switzerland) and 900 IU GONAL-f^® ^(FbM) Prefilled Pen supplied by Merck Serono S.p.A. (Bari, Italy).

Water for injection (WFI) was supplied by Galenica Senese (Siena, Italy). Reagents used for the high performance liquid chromatography (HPLC) analysis were supplied by Merck Serono S.A. – Geneva (Switzerland).

### Gonadotrophin mixing procedures

Follitropin alfa/lutropin alfa 150 IU/75 IU and follitropin alfa were mixed according to the procedures described below to provide different ratios of r-hFSH and r-hLH ranging from 3:1 to 8:1 (Table 1). In Ratio Groups 1 and 2, the r-hFSH:r-hLH ratio was 3:1, whereas the ratios of r-hFSH:r-hLH in Ratio Groups 3 and 4 were 5:1 and 8:1, respectively. A single stock solution was prepared for each Ratio Group (1–4) on study day 1 and stored at 2–8°C for the duration of the administration period. In all cases, follitropin alfa solution was added to follitropin alfa/lutropin alfa 150 IU/75 IU lyophilized powder using plastic syringes, and the final reconstituted volume was approximately 1 mL.

**Table 1:** r-hFSH and r-hLH mixing scheme*

		Follitropin alfa/lutropin alfa 150 IU/75 IU	Follitropin alfa 75 IU	Follitropin alfa 900 IU	r-hFSH:r-hLH ratio
Ratio Group 1	FSH	150 IU	75 IU in 1 mL	--	3:1
	LH	75 IU	--	--	
Ratio Group 2	FSH	150 IU	--	75 IU in 0.125 mL	3:1
	LH	75 IU	--	--	
Ratio Group 3	FSH	150 IU	225 IU in 1 mL	--	5:1
	LH	75 IU	--	--	
Ratio Group 4	FSH	150 IU	--	450 IU in 0.75 mL	8:1
	LH	75 IU	--	--	

r-hFSH, recombinant human follicle-stimulating hormone; r-hLH, recombinant human luteinizing hormone.

*Follitropin alfa was reconstituted with water for injection and then added to follitropin alfa/lutropin alfa lyophilized powder. The final reconstituted volume was always approximately 1 mL.

#### Ratio Group 1: 75 IU in 1 mL follitropin alfa (75 IU) plus follitropin alfa/lutropin alfa 150 IU/75 IU (r-hFSH:r-hLH ratio 3:1)

Step 1: A vial containing follitropin alfa 75 IU lyophilized powder was reconstituted with 1 mL of WFI using a 1 mL plastic syringe with 22 GA × 1" needle. Step 2: The vial was gently mixed until the cake was completely dissolved. Step 3: The solution was withdrawn from the vial of follitropin alfa using a 1 mL graduated plastic syringe with 22 GA × 1" removable needle. Step 4: The solution was then transferred into the vial containing follitropin alfa/lutropin alfa 150 IU/75 IU lyophilized powder. Step 5: The vial was gently mixed until the cake was completely dissolved. The needle used for withdrawing was replaced with a 27 GA × 1/2" needle and the solution was injected into a container.

#### Ratio Group 2: 75 IU in 0.125 mL follitropin alfa (900 IU GONAL-f^® ^[FbM] Prefilled Pen) plus follitropin alfa/lutropin alfa 150 IU/75 IU (r-hFSH:r-hLH ratio 3:1)

Step 1: 0.125 mL (75 IU) of follitropin alfa was injected from a 900 IU prefilled pen into a vial containing follitropin alfa/lutropin alfa 150 IU/75 IU lyophilized powder. Step 2: 0.875 mL of WFI was added to the vial containing follitropin alfa/lutropin alfa 150 IU/75 IU lyophilized powder and follitropin alfa using a 1 mL graduated plastic syringe with 22 GA × 1" removable needle. The methodology was completed according to Step 5, Ratio Group 1 (above).

#### Ratio Group 3: 225 IU in 1 mL follitropin alfa (75 IU) plus follitropin alfa/lutropin alfa 150 IU/75 IU (r-FSH:r-LH ratio 5:1)

Step 1: A vial containing follitropin alfa 75 IU lyophilized powder was reconstituted with 1 mL of WFI using a 1 mL plastic syringe with 22 GA × 1" needle. The vial was gently mixed until the cake was completely dissolved. Step 2: The solution was withdrawn from the first vial of follitropin alfa 75 IU and transferred into a second vial also containing follitropin alfa 75 IU using the 1 mL graduated plastic syringe with 22 GA × 1" removable needle. The second vial was gently mixed until the cake was completely dissolved. Step 3: The solution was withdrawn from the second vial of follitropin alfa 75 IU and transferred into a third vial containing follitropin alfa 75 IU lyophilized powder using the 1 mL graduated plastic syringe with 22 GA × 1" removable needle. The third vial was gently mixed until the cake was completely dissolved. Step 4: As much solution as possible was withdrawn from the third vial of follitropin alfa 75 IU using a 1 mL graduated plastic syringe with 22 GA × 1" removable needle and transferred into a vial of follitropin alfa/lutropin alfa 150 IU/75 IU lyophilized powder. The methodology was completed according to Step 5, Ratio Group 1 (above).

#### Ratio Group 4: 450 IU in 0.75 mL follitropin alfa (900 IU GONAL-f^® ^[FbM] Prefilled Pen) plus follitropin alfa/lutropin alfa 150 IU/75 IU (r-FSH:r-LH ratio 8:1)

Step 1: 0.75 mL (450 IU) of follitropin alfa was injected from a 900 IU prefilled pen into a vial containing follitropin alfa/lutropin alfa 150 IU/75 IU lyophilized powder. Step 2: Using a 1 mL graduated plastic syringe with 22 GA × 1" removable needle, 0.25 mL of WFI was added to the vial containing follitropin alfa/lutropin alfa 150 IU/75 IU lyophilized powder and follitropin alfa. The methodology was completed according to Step 5, Ratio Group 1 (above).

### Analytical methods

The protein content and *in vivo *bioactivity of each r-hFSH and r-hLH solution were measured after mixing.

#### Determination of the protein content of r-hFSH and r-hLH mixed solutions

The target protein content of each mixed solution was calculated using the following conversion factors: 150 IU r-hFSH corresponds with 10.8 mcg; 75 IU r-hLH corresponds with 3 mcg. Chromatography of the prepared solutions was performed on a reverse-phase-HPLC (RP-HPLC, Waters) column (source: 5RPC 4.6 × 150 mm), using a gradient of 0.1% trifluoroacetic acid/acetonitrile in 0.1% trifluoroacetic acid/water. The two subunits of r-hFSH and r-hLH were separated and the peaks relative to the beta subunits of each protein were quantified as a proportion of the beta subunits of their respective standards.

#### *In vivo *bioassay for r-hFSH biological activity

The *in vivo *bioactivity of FSH was assessed according to current US and European Pharmacopoeias by the traditional Steelman-Pohley human chorionic gonadotrophin (hCG) augmentation assay (1953), which measures ovarian hypertrophy following administration of exogenous FSH (in combination with hCG) to immature female rats [5].

Animal groups of five, 21-day-old female Sprague-Dawley rats (Harlan-Nossan, Italy) weighing 42–52 g were used for each bioassay. The rats were injected with both the follitropin alfa/lutropin alfa mixed solution and hCG (Ovitrelle^®^) for 3 days; they received one dose on day 1 and two doses on days 2 and 3. On day 4, the rats were sacrificed and their ovaries were removed, excised of fat and connective tissue and weighed. Each sample was tested in triplicate (using three independent replicates). The internal Merck Serono S.A. – Geneva Standard was previously calibrated against the National Institute for Biological Standard and Controls (NIBSC) International Standard (recombinant follicle stimulating hormone, IS 92/642).

#### *In vivo *bioassay for r-hLH biological activity

The *in vivo *bioactivity of LH was assessed according to US and European Pharmacopoeias following the Van Hell seminal vesicle weight gain bioassay [6].

Animal groups of five, 21-day-old male SPF Sprague-Dawley rats (Harlan-Nossan) weighing 42–52 g were used. Rats were injected with one subcutaneous daily dose (0.5 mL) of the follitropin alfa/lutropin alfa 150 IU/75 IU and follitropin alfa mixed solution for 4 days. On day 5, 96 hours after the first administration, the animals were sacrificed and the seminal vesicles were removed and weighed. Each sample was tested in triplicate. The internal Merck Serono S.A. – Geneva Standard was previously calibrated against the NIBSC International Standard (recombinant luteinizing hormone, IS 71/264).

#### Animal care

Animal studies were authorized by the Italian Health Authority (Ministero della Salute) in accordance with the government decree D.L. 116/92 and the 86/609/CEE directive. All animals were kept in 12 hours of light and 12 hours of dark conditions, and each experimental animal group was housed in separate cages. Rats were given access to food and water *ad libitum *throughout the study.

## Results

Follitropin alfa/lutropin alfa 150 IU/75 IU lyophilized powder could be mixed successfully with follitropin alfa 75 IU FbM solution that was either reconstituted from lyophilized powder or injected directly from the GONAL-f^® ^(FbM) Prefilled Pen. Using these commercially available formulations, it was possible to create solutions with ratios of r-hFSH and r-hLH in the range from 3:1 to 8:1. Furthermore, no practical issues such as needle bending were experienced during the addition of follitropin alfa solution from the prefilled pen to follitropin alfa/lutropin alfa 150 IU/75 IU lyophilized powder.

The r-hFSH and r-hLH content and *in vivo *bioactivity of mixed solutions of follitropin alfa/lutropin alfa 150 IU/75 IU lyophilized powder and follitropin alfa are presented in Table 2. The measured protein content of r-hFSH and r-hLH corresponded closely with the target amount; the coefficient of variation ranged from 0.3 to 1.2% for r-hFSH and from 1.1 to 2.9% for r-hLH. The measured bioactivity of r-hFSH and r-hLH also corresponded closely with the target amount. Thus, both the measured protein content and *in vivo *bioactivity of r-hFSH and r-hLH in Ratio Groups 1–4 corresponded favourably with the target amounts.

**Table 2:** r-hFSH and r-hLH protein content and *in vivo *bioactivity of mixed solutions of follitropin alfa and lutropin alfa

		Protein content			Bioactivity*	
		Target (mcg/mL)	Measured (mcg/mL)	CV (%)	Target (IU/mL)	Measured (IU/mL)
Ratio Group 1 (n = 15)	r-hFSH	16.2	16.5	1.2	225	216
	r-hLH	3.0	3.4	1.7	75	77
Ratio Group 2 (n = 15)	r-hFSH	16.2	17.4	0.9	225	221
	r-hLH	3.0	3.5	2.9	75	76
Ratio Group 3 (n = 15)	r-hFSH	27.0	26.9	1.1	375	339
	r-hLH	3.0	3.5	1.1	75	73
Ratio Group 4 (n = 15)	r-hFSH	43.2	45.4	0.3	600	568
	r-hLH	3.0	3.5	2.9	75	77

CV, coefficient of variation; r-hFSH, recombinant human follicle-stimulating hormone; r-hLH, recombinant human luteinizing hormone.

The target protein content was calculated using the following conversion factors: 150 IU r-hFSH corresponds with 10.8 mcg; 75 IU r-hLH corresponds with 3 mcg.

*All bioactivity measurements fell within the 80 – 125% of target, as specified by the United States Pharmacopeia for product labelling [20].

## Discussion

Infertility treatment protocols are individualized according to a variety of factors, including the patient's age and diagnosis, ovarian reserve and any co-administered medications. Owing to the complexity of treatment protocols, patients prefer to use the fewest possible number of steps to prepare each injection [7]. Accordingly, the ability to vary the FSH to LH dose ratio in a single injection is important for both physicians and patients.

The pharmacokinetics of follitropin alfa and lutropin alfa administered individually or mixed together was first reported by le Cotonnec *et al*. [8-13]. The authors found no evidence of pharmacokinetic or pharmacodynamic interactions between follitropin alfa and lutropin alfa. More recently, it has been demonstrated that r-hLH and r-hFSH can be mixed together in the same syringe without compromising the bioactivity of the gonadotrophins [14], and administered successfully to patients [15]. Purified urinary gonadotrophin products can also be reconstituted and mixed together in a single syringe without affecting the FSH or LH bioactivity [16].

The present study was conducted to assess the feasibility of reconstituting follitropin alfa/lutropin alfa 150 IU/75 IU lyophilized powder using currently marketed formulations of follitropin alfa FbM solution. The results demonstrate that follitropin alfa FbM either as 75 IU lyophilized powder or multi-dose solution from a prefilled pen may be mixed with follitropin alfa/lutropin alfa 150 IU/75 IU lyophilized powder to produce solutions with ratios of follitropin alfa to lutropin alfa from 3:1 to 8:1.

Regulatory bodies designate that the Steelman-Pohley and Van Hell seminal vesicle weight gain *in vivo *bioassays are used to determine the gonadotrophin content of commercial FSH and LH products [5,6]. However, the inherent lack of precision of these bioassays is well recognized [17-19]. To allow for this variability, it is specified in the United States Pharmacopeia, that gonadotrophin formulations should contain between 80% and 125% of the hormones listed on the product label [20].

In this study, the bioactivity of FSH and LH as assessed by the established *in vivo *bioassays fell well within these bounds. Thus, the bioactivity of FSH and LH was unaffected after mixing using plastic syringes and following the methodology described. The follitropin alfa and lutropin alfa protein content of the mixed solutions was also assessed by an accurate RP-HPLC assay, which confirmed the expected recoveries.

The flexibility in dosing ratios of follitropin alfa and lutropin alfa afforded by mixing facilitates further individualization of therapy according to each patient's individual gonadotrophin requirements.

## Conclusion

Follitropin alfa can be mixed accurately with follitropin alfa/lutropin alfa 150 IU/75 IU lyophilized powder to enable physicians to administer different ratios of r-hFSH and r-hLH in the IU range from 3:1 to 8:1. Mixing of recombinant formulations is expected to offer flexibility for patients receiving follitropin alfa and lutropin alfa for ART protocols in clinical practice.

## Competing interests

Rita Agostinetto is an employee of Merck Serono S.p.A., Ardea, Italy. She works in the Pharmaceutical Development-Biotechnology Products Department and has experience of the development of injectable proteins, and a particular interest in gonadotrophins.

## Authors' contributions

RA contributed to the study design, wrote the first draft of the manuscript, and read and approved the final draft.
